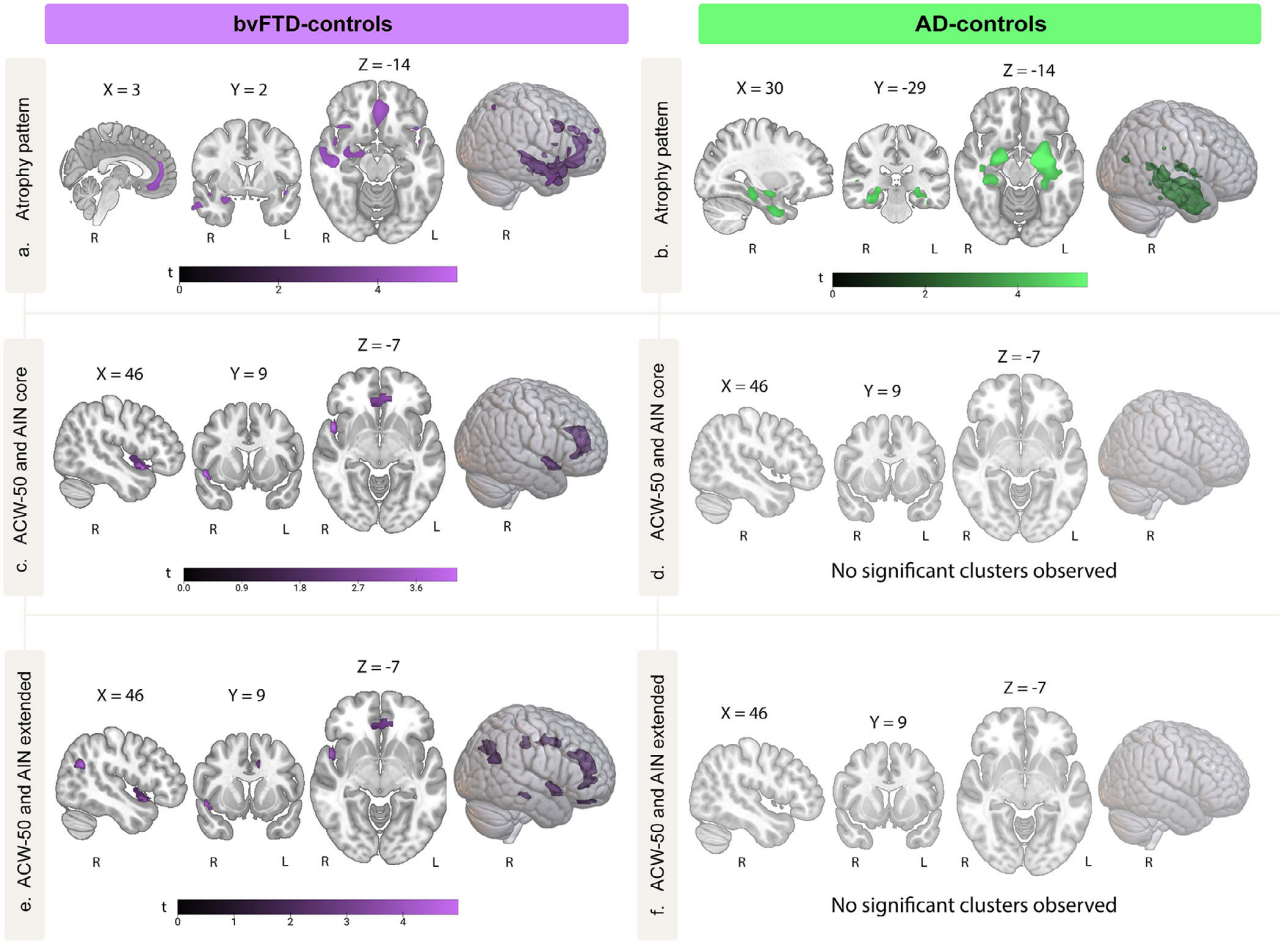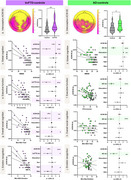# Altered spatiotemporal brain dynamics of interoception in behavioural‐variant frontotemporal dementia

**DOI:** 10.1002/alz70862_109837

**Published:** 2025-12-23

**Authors:** Jessica L Hazelton, Gabriel Della Bella, Pablo Barttfeld, Martin Dottori, Raul Gonzalez‐Gomez, Joaquín Migeot, Sebastian Moguilner, Agustina Legaz, Hernan Hernandez, Pavel Prado, Jhosmary Cuadros, Marcelo Adrian Maito, Matias Fraile‐Vazquez, María Luz González Gadea, Yasir Çatal, Bruce L. Miller, Olivier Piguet, Georg Northoff, Agustin Ibanez

**Affiliations:** ^1^ Cognitive Neuroscience Center (CNC), Universidad de San Andrés, Buenos Aires, Buenos Aires Argentina; ^2^ The University of Sydney, Brain and Mind Centre, Sydney, NSW Australia; ^3^ Latin American Brain Health Institute (BrainLat), Universidad Adolfo Ibañez, Santiago Chile; ^4^ Instituto de Investigaciones Psicológicas (IIPsi, CONICET‐UNC), Facultad de Psicología, Universidad Nacional de Córdoba, Córdoba, Córdoba Argentina; ^5^ Facultad de Matemática Astronomía y Física (FaMAF), Universidad Nacional de Córdoba, Córdoba, Córdoba Argentina; ^6^ Latin American Brain Health Institute (BrainLat), Universidad Adolfo Ibáñez, Santiago, Región Metropolitana de Santiago Chile; ^7^ Global Brain Health Institute (GBHI), University of California San Francisco (UCSF); & Trinity College Dublin, Dublin, Leinster Ireland; ^8^ Escuela de Fonoaudiología, Facultad de Odontología y Ciencias de la Rehabilitación, Universidad San Sebastián, Chile, Santiago, Región Metropolitana de Santiago Chile; ^9^ Advanced Center for Electrical and Electronic Engineering (AC3E), Universidad Técnica Federico Santa María, Valparaíso, Yaracuy Venezuela (Bolivarian Republic of); ^10^ Universidad Nacional Experimental del Táchira, San Cristóbal, Táchira Venezuela (Bolivarian Republic of); ^11^ Life Span Institute, University of Kansas, Lawrence, KS USA; ^12^ National Scientific and Technical Research Council (CONICET), Buenos Aires, Buenos Aires Argentina; ^13^ Mind, Brain Imaging and Neuroethics, Institute of Mental Health Research, University of Ottawa, Ottawa, ON Canada; ^14^ Global Brain Health Institute, University of California, San Francisco, San Francisco, CA USA; ^15^ Memory and Aging Center, Weill Institute for Neurosciences, University of California, San Francisco, San Francisco, CA USA; ^16^ The University of Sydney, School of Psychology, Sydney, NSW Australia; ^17^ Mental Health Center, Zhejiang University School of Medicine, Hangzhou, Zhejiang China; ^18^ Center for Cognition and Brain Disorders, The Affiliated Hospital of Hangzhou Normal University, Hangzhou, Zhejiang China

## Abstract

**Background:**

Dysfunctional allostatic‐interoception, altered processing of bodily signals in response to environmental demands, occurs in behavioural‐variant frontotemporal dementia (bvFTD) patients. Previous research, however, has focused on static measures of interoception (e.g., heart‐evoked potential, HEP). These measures do not capture the dynamic nature of interoception, unlike intrinsic neural timescales. Intrinsic neural timescales refers to the temporal durations over which information is processed within the spatiotemporal hierarchy of the brain, with shorter timescales representing more rapid processing. We hypothesised that longer intrinsic neural timescales of interoception would occur in bvFTD patients, evidencing dysfunctional allostatic‐interoception.

**Method:**

One‐hundred and twelve participants (31 bvFTD patients, 35 Alzheimer’s disease patients, AD and 46 healthy controls) completed a well‐validated task measuring cardiac‐interoception and exteroception. Simultaneous EEG and ECG were recorded. Intrinsic neural timescales were measured via the autocorrelation window (ACW) of broadband EEG signals from each heartbeat and a time‐lagged version of itself. Spatiotemporal clustering analyses identified clusters with significant between‐group differences in each condition. HEP modulation analyses were also conducted and covaried for to investigate potential relationships between HEP and ACW. Voxel‐based morphometry was used to target the allostatic‐interoceptive network. Neuropsychological tests of cognition and social cognition were assessed.

**Result:**

In bvFTD patients, longer interoceptive‐ACWs than controls were observed in the bilateral fronto‐temporal and parietal regions (Figure 1). In AD patients, longer interoceptive‐ACWs than controls were observed in central and occipitoparietal brain regions (Figure 1). No differences were observed during exteroception. In bvFTD patients only, longer interoceptive‐ACW was linked to worse sociocognitive performance. Our interoceptive‐ACW results, remained the same when accounting for HEP modulation. Structural neural correlates of interoceptive‐ACW in bvFTD involved the anterior cingulate, insula, orbitofrontal cortex, hippocampus, and angular gyrus (Figure 2). No structural differences emerging in AD.

**Conclusion:**

Our findings suggest a core allostatic‐interoceptive deficit occurs in people with bvFTD, captured by altered brain dynamics of intrinsic neural timescales. In AD, it is possible that a more generalised disruption of brain oscillation occurs. Altered interoceptive intrinsic neural timescales may provide a neurobiological mechanism underpinning the complex behaviours observed in bvFTD patients. Our findings support synergistic models of brain disease and can inform clinical practice.